# The Analysis of Erosive Wear Resistance of WC-Co Carbides Obtained by Spark Plasma Sintering Method

**DOI:** 10.3390/ma14237326

**Published:** 2021-11-30

**Authors:** Joanna Wachowicz, Tomasz Dembiczak, Grzegorz Stradomski, Zbigniew Bałaga, Joanna Jasińska, Dariusz Rydz, Jacek Wilkowski, Marcin Dyner

**Affiliations:** 1 Department of Mechanical Processing of Wood, Institute of Wood Sciences and Furniture, Warsaw University of Life Sciences, Nowoursynowska Street 166, 02-787 Warsaw, Poland; jacek_wilkowski@sggw.edu.pl; 2 Faculty of Science and Technology, Jan Dlugosz University in Czestochowa, Armii Krajowej Street 13/15, 42-200 Czestochowa, Poland; t.dembiczak@ujd.edu.pl (T.D.); joanna.jasinska@ujd.edu.pl (J.J.); m.dyner@ujd.edu.pl (M.D.); 3 Faculty of Production Engineering and Materials Technology, Czestochowa University of Technology, Armii Krajowej Street 19, 42-201 Czestochowa, Poland; gstradomski@wip.pcz.pl (G.S.); balaga.zbigniew@wip.pcz.pl (Z.B.); rydz.dariusz@wip.pcz.pl (D.R.)

**Keywords:** sintering, spark plasma sintering, cemented carbides WC-Co, powder metallurgy, erosive resistance

## Abstract

WC-Co (tungsten carbide-cobalt) composites are widely used in industry, wear-resistant parts, and cutting tools. As successful tool materials, WC-Co carbides are widely applied in metal cutting, wear applications, chipless forming, stoneworking, wood, and plastic working. These materials are exposed to severe solid particle erosion by sand particles, such as in the wood industry. During the production of furniture with HDF (High Density Fibreboard), MDF (Medium Density Fibreboard), or OSB (Oriented Strand Board), there are observed problems with tool erosion. Contamination, mainly of the HDF by sand, is quite often, which is why all tools used for the machining of such materials are exposed to erosion by sand particles. Although many studies have been performed on the erosion of various metals, and erosion models exist to predict their erosion behavior, the issue is still relevant. The aim of the study was to determine the effect of grain size (submicron, ultrafine) and the manufacturing technology (SPS—Spark Plasma Sintering, conventional) used on the erosive properties of WC-Co sintered carbides. Sinters produced by the SPS method with different sizes of WC grains and commercial samples were used for the tests. Ten two-hour cycles were carried out under medium conditions of quartz sand and quartz sand with 10% SiC added. Used samples were characterised using scanning electron microscopy (SEM) and roughness was determined. Furthermore, erosion studies allowed individuating a wear mechanism as well as the possibility to foresee cutting performance in prospective application.

## 1. Introduction

WC-Co (composite of tungsten carbide and cobalt) carbides, known for their attractive properties, are widely used in the tooling industry. These properties include: high hardness, abrasion resistance, and relatively high resistance to brittle fracture. The mechanical and cutting properties of carbides depend on chemical composition, grain size, and manufacturing technology. As the relative volume of cobalt increases, the bending strength of the carbides increases, while the hardness and resistance to cutting wear decrease [1,2]. Despite being known for nearly 100 years, they are still of great interest to researchers. The researchers are making attempts to increase hardness while maintaining high strength. One of the methods is obtaining WC-Co nanocomposites [3,4,5,6,7,8,9,10,11]. There has been an increasing trend, recently, towards research on solid particle erosion wear; one of the types of surface damage, where eroding particles, of different dimensional sizes under various impact velocities and impingement angles, strike and affect the top surfaces of materials, thereby leading to material losses and functional variations [12]. WC-Co tool composites are exposed to severe solid particle erosion by sand particles, such as in the wood industry. Although many studies have been performed on the erosion of various metals, and erosion models exist to predict their erosion behavior, comprehensive studies and models on the erosion of WC-Co composites are not available [13]. The work of [14] presents a study on the performance of innovative coatings for steel cutting tools (HSS18) in machining wood. Tribological and erosion characterization suggested the most important features to be the right combination of hardness, toughness, and adhesion between coating and substrate. However, when the tool surface was too brittle, this caused a premature failure of the cutting edge. The results of the machining tests showed substantial agreement with this pattern, since wood is a highly abrasive and non-homogeneous material, with a high amount of inclusions of hard particles.

Powder metallurgy is a commonly used method to produce various types of materials. The field of powder metallurgy offers many opportunities for the development and modernization of this technology. Sintering is one of the basic technological processes used in powder metallurgy. The sintering technology involves the transformation of compressed powder into a solid material, which may be accompanied by shrinkage. Conventionally, pre-consolidated powders are sintered in resistance or induction heating furnaces, in a reducing or inert atmosphere [15,16,17,18,19,20].

In recent years, innovative powder material consolidation technologies of the SPS/FAST (Spark Plasma Sintering/Field Assisted Sintering Technology) type have been used to improve certain properties (such as density, hardness). SPS ensures to attain homogenous highly dense sintered compacts faster, at a lower temperature, and with a finer microstructure, than conventional sintering methods [21]. In these methods, in contrast to the conventional sintering methods, pulses of electric current are used to heat the powder, which creates specific sintering conditions. The applied method of heating the powder with simultaneous pressing allows sintering at a lower temperature and in a shorter time, than in conventional processes. In addition, SPS/FAST methods have the advantage of economic considerations: mainly the energy efficiency (in these methods only the sintered material is heated, with no energy lost to radiation and convection). It is estimated that power consumption is between 1/3 and 1/5 compared to consolidation techniques, such as: hot sintering (HP) and hot isostatic pressing (HIP). However, SPS/FAST methods also have some limitations. They are associated with the size and shape of the products, and the uniformity of the temperature field in the sample volume, as well as the occurrence of stresses when cooled too quickly [22,23,24,25,26,27,28].

The exact mechanism of sintering, as well as the role of pulsed current in electric field-assisted methods, despite a number of studies, are currently not fully explained. There are two major theories on SPS mechanism in discussion [29,30,31]. One is with effect of spark and plasma at the initial stage; the other is a lack of an appearance of spark and plasma during the entire SPS process. It should be noted that different phenomena are observed in both theories for the sintering of metals and ceramic materials. The basic mechanism of SPS is based on the following steps, as described by Tokita [21]: (1) spark plasma, (2) spark impact pressure, (3) Joule heating, and (4) an electrical field diffusion effect. In the SPS process, their surface is more cleaned and activated, compared to conventional sintering methods, due to the pulses of direct current flowing through the powder particles. Therefore, high quality sinters are obtained at a lower temperature and in a shorter time [30,31,32,33].

The aim of this paper was to determine the influence of used technology on the erosive resistance of WC-Co sintered materials. The processes of the Spark Plasma Sintering and conventional methods were analyzed. For the erosion tests, two sets (by the SPS method) of samples with variant WC particle size (approx. 400 nm and 1 µm) were made and used as commercial specimen.

## 2. Materials and Methods

**Figure 1:**
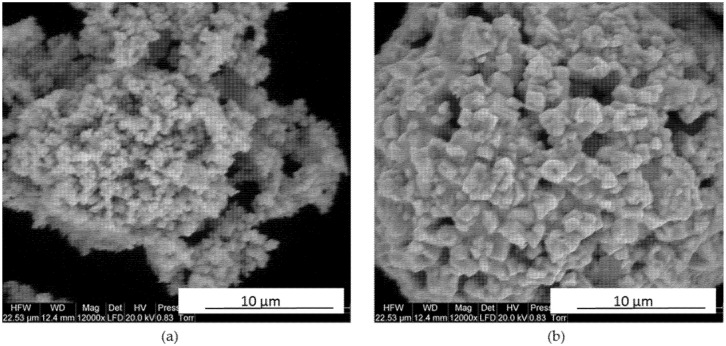
SEM images of WC-Co powders used to prepare sinters: (**a**) primary particle size of WC about 1 μm (submicron); (**b**) primary particle size of WC approx. 400 nm (ultrafine) [34].

The research carried out is a continuation of the study [34]. Mixed WC and Co powders were used in the tests. The powders used differed in the size of the primary WC particles. The particle size of the first powder was about 1µm (submicron), while the second was about 400 nm (ultrafine). Microscopic observations (using SEM-FEI QUANTA 200, FEI, Hillsboro, OR, USA) of the 400 nm particle-size powder showed spherical agglomerates of about 20 μm. In contrast, WC powder with a particle size of about 1 μm showed a spherical shape. About 1 μm showed an irregular shape, with only some powder particles forming irregular agglomerates of different sizes (Figure 1).

The WC-Co carbide sintering process was carried out using a Spark Plasma Sintering (SPS) device from ITME (Warsaw, Poland), in two stages. The degassing stage was carried out at temperature of about 600 °C for 3 min, increasing the load from 30 to 50 MPa in the second minute. The second stage, the sintering itself, was carried out at 1170 °C for 5 min. The sintered samples had a diameter of 20 mm and a height of 2.5 mm. The density of samples was measured using the Archimedes method, with a Mettler Toledo ME204 Balance (Langacher Greifensee, Switzerland). Hardness measurements were carried out using the Vickers method, in accordance with the PN-EN ISO 6507-1: 2007 standard, using an FM-700 hardness tester (Future-Tech Corp FM-700, Tokyo, Japan) at a load of 294.2 N. The basic properties of the sintered materials are summarised in Table 1. The hardness results obtained had similar values over the entire cross-section of the samples. This indicates uniform sintering of the samples in the entire volume. The maximum value of the stress intensity factor was obtained for the commercial samples. Despite the lower content of cobalt (4.5 wt%), the ultrafine sinters showed a slightly lower hardness compared to sinters containing 6% Co. The reason for this may be the growth of WC grains after sintering, which was observed for ultrafine sintering [34].

**Figure 2:**
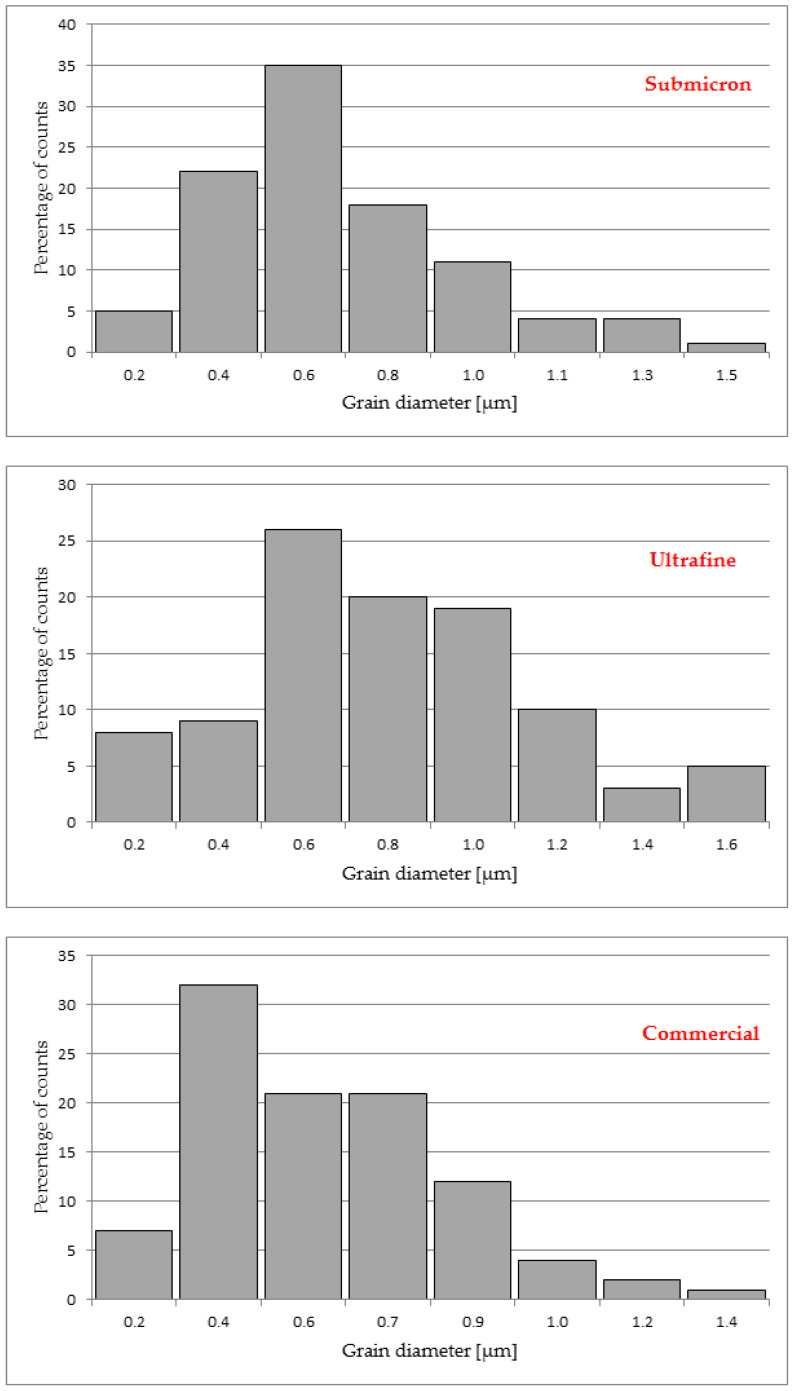
Distribution of grain sizes after the sintering process of the WC-Co composite, presented in the histogram.

A histogram shows the grain sizes after the sintering process, using an image analysis programme, and the results are shown in Figure 2.

**Figure 3:**
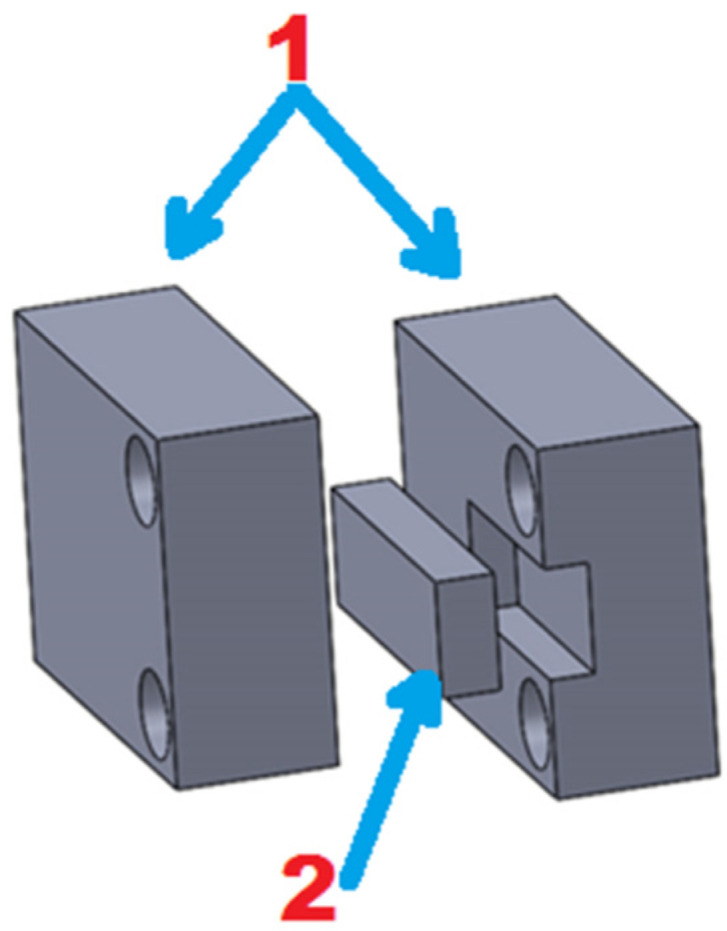
Sample fixing holder: 1—mounting fixtures, 2—sample.

Samples of WC-Co sinters, produced by Spark Plasma Sintering from submicron and ultrafine powders, were prepared for erosion wear testing. In addition, erosion wear tests were carried out for a WC-Co commercial plate from Baildonit (Katowice, Poland). The samples were cut, using a wire-cutting machine from FANUC (Oshino, Japan), to a dimension of 11 mm × 4 mm × 2 mm, and, additionally, mounting fixtures made of 316 L steel (shown in Figure 3) were made to fix the specimens in the rotating disk of the erosion simulator.

**Figure 4:**
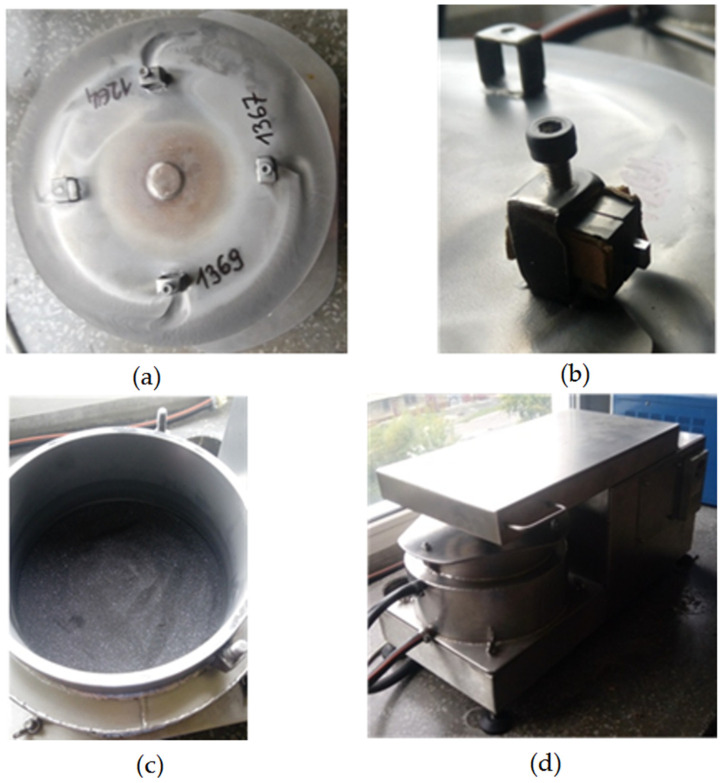
Erosion simulator: (**a**) disk to which the samples are attached; (**b**) sample fixing; (**c**) container with erosive medium filled; (**d**) view of erosion simulator prepared for testing.

The erosion wear test was carried out at the Department of Production Engineering and Materials Technology, Czestochowa University of Technology (Czestochowa, Poland). The view of the erosion test rig is shown in Figure 4. The samples were degreased in methyl alcohol (CH_3_OH), using an ultrasonic cleaner for 5 min, before testing. The samples were then weighed using an OHAUS PX125D analytical balance (Parsippany, NJ, USA), with a measurement accuracy of 0.00001 g.

The surface of cut specimens was analysed using an SEM Phenom XL microscope (Waltham, MA, USA) in the initial state and after 10 cycles of erosive wear (per medium).

Analysis of the change in geometric structure of the surface of the sintered samples, in the initial state and after 10 cycles, was carried out using a Sensofar S Neox 3D optical profilometer (Barcelona, Spain). The technologies used in the device enable high-resolution image capture and detailed observation as well as automatic measurement of surfaces of any roughness. Roughness measurements were carried out three times in the same area defined per sample, and the results presented were averaged.

## 3. Results and Discussion

**Figure 5:**
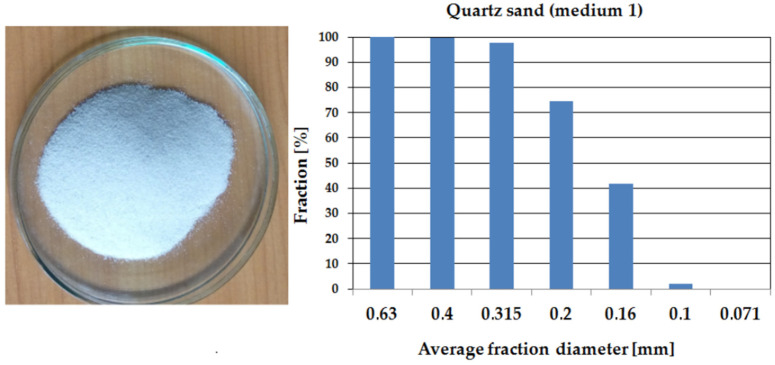
Particle size distribution of quartz sand (medium 1) presented as a histogram.

**Figure 6:**
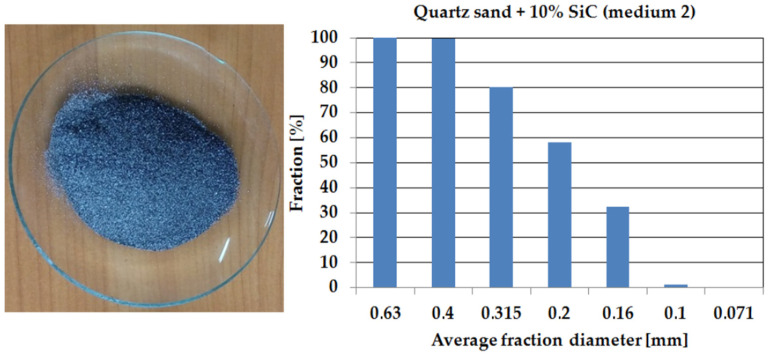
Particle size distribution of quartz sand + 10% SiC (medium 2) presented as a histogram.

Media used in the erosion wear study were quartz sand (medium 1, HV = 10,781 MPa) and quartz sand with the addition of 10% SiC (medium 2, HV = 24,000 MPa). Sieve analysis, performed to show similarities and differences in the nature of the erosive media, was carried out using sieve shaker model LPzE-2e (Marcyporeb, Poland). To carry out the sieve analysis, 100 g of sand was used and the set of sieves was as follows: 0.63, 0.4, 0.315, 0.2, 0.16, 0.1, and 0.071 mm; shaker working time was 5 min. Figure 5 and Figure 6 show the results obtained for each media.

The sieve analysis shows that medium 1 is characterised by a particle size distribution approximately 15% larger than medium 2.

**Figure 7:**
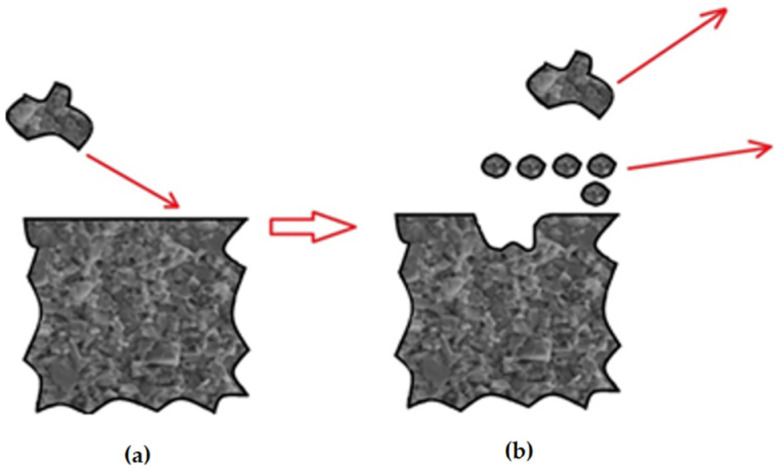
Erosion caused by an impact of solid particles on the surface: (**a**) surface before impact of the medium particle; (**b**) after impact of the particle. Own study based on [35].

In general, the erosion test in a medium tank involves chaotically moving solid hard particles, which hit the surface of the target material at different velocities and different impact angles, from 0 to 90°. When the impact angle is parallel to the test surface or close to 0°, the main wear mechanism is abrasion rather than erosion, and the damage produced is mainly micro-erosion, wedge formation, and micro-cutting [35], according to the scheme shown in Figure 7.

**Figure 8:**
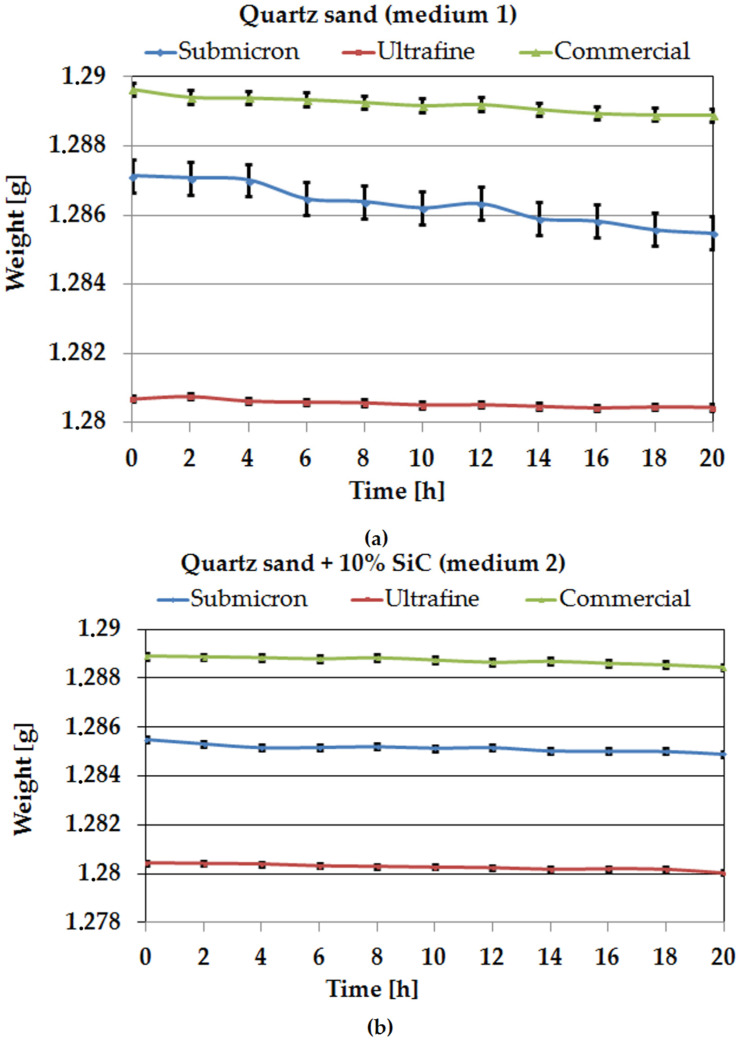
Cumulative erosion expressed as mass loss as a function of testing time for submicron, ultrafine, and commercial samples: (**a**) medium 1; (**b**) medium 2.

In the erosion wear tests, fatigue wear was observed on all surfaces of the samples as a result of chipping and peeling of various sizes (Figure 8). Several variations in the slope were observed in the test analysis, indicating that the rate of erosion wear depends, in particular, on the type of WC-Co sinter and the erosion medium used. During the test cycles, the percentage of material loss was highest for the quartz sand medium. This indicates that the number of impacts of large particles and their concentration was higher; however, a higher concentration of smaller particles that can “penetrate” the tested material causes its erosion. This can be deduced from the effect of the SiC addition in medium 2, where the amount of large hard particles is 10%.

**Figure 9:**
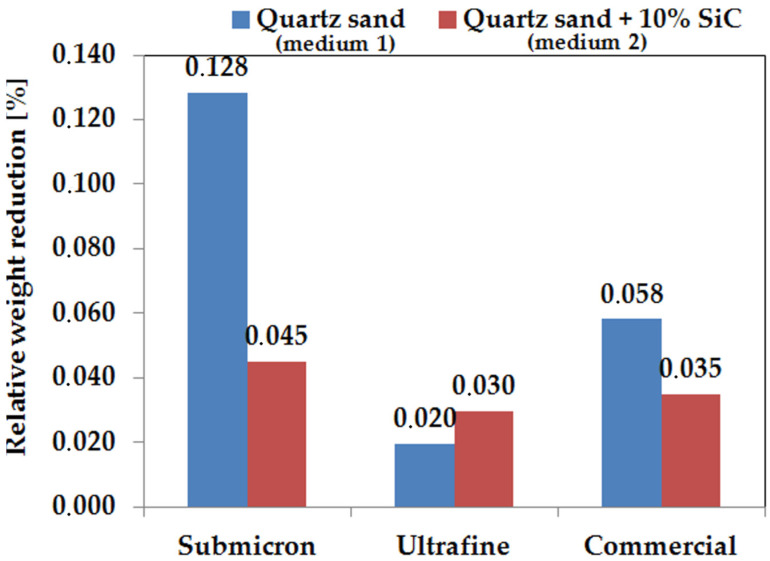
Graph of relative weight loss after 10 cycles, with a distance of 14,695.2 m used for medium 1 and medium 2.

The average value of relative weight loss is shown in Figure 9. The total mass loss after 20 h for the erosion medium, which was quartz sand, varied depending on the WC-Co sinter produced. The ultrafine sample showed the lowest wear of 0.020% relative to the initial mass, and a relative mass loss of 0.096% was observed for the commercial sample, while the submicron sample showed a mass loss of 0.139%. After the erosion tests, no significant erosion wear was observed; the results obtained are in the range of less than 1%. This shows that the WC-Co sinter produced by spark plasma sintering (SPS) technology and the commercial sinter produced by conventional sintering are characterised by high relative density and low pore volume in the sinter volume. However, it should be emphasised that the materials studied are designed for long-term tribological study, which the authors have already presented in a previous study [34]. The friction path under erosive conditions for each medium was over 14 km, and up to about 1% loss was already observed during this time, which indicates that erosion is an important factor in the wear of such hard materials.

It was observed that for medium 2, which was quartz sand with the addition of 10% SiC, the erosive wear of the tested samples is lower than for the variant with medium 1. This is due to the fact that, as a result of the collision, sand particles are degraded and fragmented. The fine particles penetrate the gaps between the WC and have an erosive effect on the relatively soft Co phase. This effect leads to the appearance of furrows and, eventually, to the loss of whole hard WC particles, which is evident from the weight loss of the samples. The obtained results of relative weight loss for each tested sample are in the range below 0.5%. This is all the more interesting, as previous studies by the authors [36] related precisely to the use of erosion medium with SiC additives, indicate that it is highly aggressive. However, the earlier studies focused mainly on solid or relatively soft materials, which resulted in the occurrence of different type of damage phenomena. In the case of the materials with a hardness of 1632–1736 HV presented here, the effects of the medium are different.

**Figure 10:**
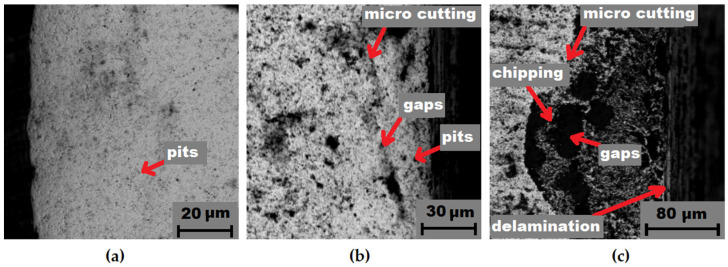
Erosion wear after 10 cycles of a WC-Co submicron sinter sample: (**a**) initial condition; (**b**) medium 1; (**c**) medium 2.

**Figure 11:**
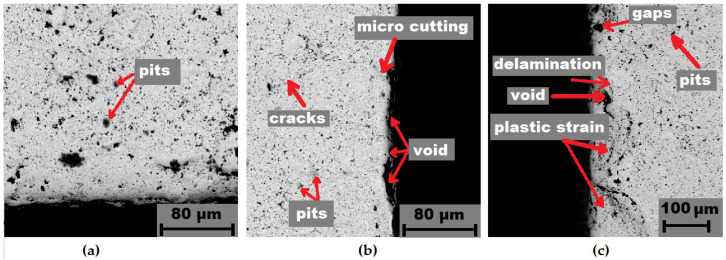
Erosion wear after 10 cycles of a WC-Co ultrafine sinter sample: (**a**) initial condition; (**b**) medium 1; (**c**) medium 2.

**Figure 12:**
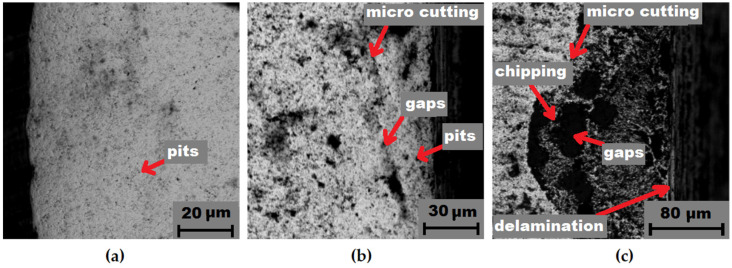
Erosion wear after 10 cycles of a WC-Co commercial sample: (**a**) initial condition; (**b**) medium 1; (**c**) medium 2.

Further on, after erosive wear the samples were subjected to a detailed evaluation of the working surface (subjected to erosion), using SEM microscopy; the results are presented in Figure 10, Figure 11 and Figure 12. The characteristics of the cross sections showed that several overlapping wear mechanisms are visible. It was found that the main mechanisms responsible for the erosive behavior of the studied WC-Co sinters were chipping, delamination micro cutting, plastic strain, cracks, void, gaps, and pits.

At larger impact angles, the material surface is deformed, displaced, or removed from the impact site, where the particle can penetrate the surface depending on the impact energy [37]. For many materials, such as soft metals and polymers, embedment of the most common erosion phenomena occurs at normal impact angles. However, for ceramics, it might be increased wear. At impact angles approximately normal to the surface under test, the displacement and deformation of the material reach a maximum, and a significant amount of material can easily be ejected from the target surface, by massive particles, as seen in Figure 10c. However, different erosion mechanisms usually act simultaneously, depending on kinetic energy of particles, the angle of impact, and the local properties of the target material, such as hardness and density [38]. An example of a highly deformed surface section is shown in Figure 11c. In this case, it is clearly visible that “large”, hard SiC particles hitting the hard surface of the specimen are resiliently deflected so that the degree of damage from erosion is less [39]. This is interesting, in that for traditional materials, such as low carbon bainitic steels, the more aggressive medium, i.e., which contains a higher number of hard particles, acts much more strongly [40]. It is obvious that if the bonding material is so highly eroded (groove and binder), there will be a significant weight loss and, thus, an erosion trace.

Moreover, in order to verify the erosion resistance of the materials studied, changes in the geometrical structure of the surface were assessed. The parameters were assessed in the initial state and after a full erosion resistance test. For this purpose, the Sensofar S Neox 3D surface profilometer (Barcelona, Spain) was used. The technologies used in the device enable high-resolution image capture and detailed observation as well as automatic measurement of surfaces of any roughness. The measurement was carried out three times in the same area defined per sample, and the results presented were averaged.

The following stereometric parameters were considered when analysing the surface topography: S_a_—arithmetic mean surface height; S_z_—maximum surface height being the sum of the height of the highest profile height S_p_ and the depth of the lowest profile depression S_v_ in the defining area; S_v_—depth of the lowest depression in the defining area; S_p_—highest surface height in the defining area; S_ku_—clustering coefficient of the distribution of surface topography (ordinates) heights; S_sk_—skewness coefficient of the distribution of surface topography (ordinates) heights; and S_q_—mean square deviation of surface roughness heights from the reference plane.

The results of the study of geometric structure parameters in the initial state are presented in Table 2.

The results of the geometric structure parameters, after all cycles of the erosion resistance test with medium 1, are presented in Table 3.

The results of the geometric structure parameters, after all cycles of the erosion resistance test with medium 2, are presented in Table 4.

**Figure 13:**
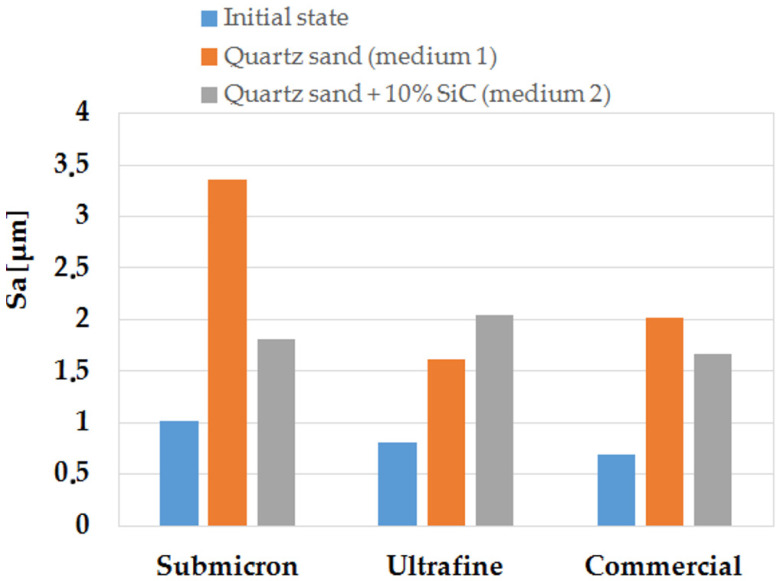
Arithmetic mean surface height (S_a_) of the tested materials in the initial state, after erosion testing in medium 1 and medium 2.

Figure 13 shows a graph of the parameter S_a_ of the tested surfaces in the initial state, after erosion testing in medium 1 and medium 2.

**Figure 14:**
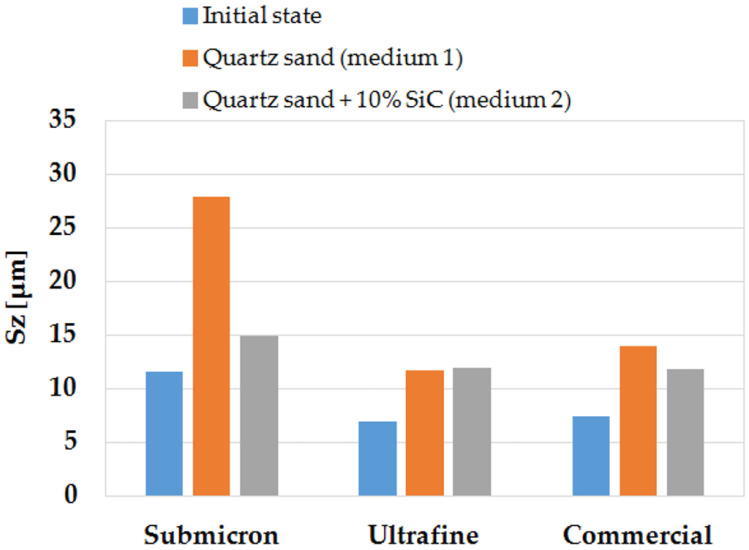
Parameter of maximum surface height (S_z_) of the tested materials in the initial state, after erosion testing in medium 1 and medium 2.

Figure 14 shows the maximum surface height parameter (S_z_), which is the sum of the height of the highest profile height (S_p_) and the depth of the lowest profile depression (S_v_) in the area defining the tested surfaces in the initial state, after erosion testing in medium 1 and medium 2.

The obtained stereometric parameters indicate that the materials studied—before the erosion resistance test—had similar surface topography characteristics. The WC-Co commercial sinter had the lowest surface roughness of S_a_ = 0.69 µm, while the sinter produced by spark-plasma sintering (SPS) had a slightly higher surface roughness; the ultrafine sinter had a roughness of S_a_ = 0.81 µm, and the submicron sinter had a roughness of S_a_ = 1.02 µm, respectively.

As a result of the tests carried out using medium 1, the roughness parameter Sa for all the samples in the defining area essentially increased by more than 100%. In addition, the maximum surface height expressed by the S_z_ parameter increased significantly for the submicron sinter by 140%, the ultrafine sinter by 67%, and the commercial sinter by 90%. The skewness coefficient of the materials both in the initial state and after erosion testing was negative, and it did not change significantly due to erosion. The concentration coefficient of the surface topography height distribution (S_ku_) of all samples was positive. After testing, the submicron and commercial sinter samples achieved a change in kurtosis towards a normal distribution; only the S_ku_ for the ultrafine sinter sample increased the positive value and was 4.86.

Analysing the results of the geometrical structure parameters after tests with medium 1 and medium 2, it can be observed that, also in this case, an increase in the parameter S_a_ is visible in relation to the initial state; however, for the sample made of submicron sinter, it is significantly smaller than in medium 1. The increase in S_a_ for the commercial sinter sample was also smaller. It was only observed that the ultrafine sinter sample, after testing in medium 2, had a higher S_a_ parameter, both relative to the initial state and to the working surface in medium 1.

Analysis of geometric structure parameters of the surface after erosion tests in both media and the results of mass loss for individual samples shows that the phenomenon of erosive wear proceeds differently for both media. For medium 2, which was quartz sand with the addition of 10% SiC, erosive wear in the form of mass loss of samples is lower than for the variant with medium 1 for quartz sand, plus the change in roughness parameters is smaller. This is due to the different particle size of the erosion medium.

## 4. Conclusions

Two groups of WC-Co sinters produced by the SPS method and commercial tools of very similar chemical composition and mechanical properties were used in the study. The conducted research is a continuation of earlier studies by the authors [34], in which wear and cutting edge wear were analysed during the cutting of an 18 mm thick chipboard.

Erosive wear tests were carried out for two media (quartz sand and quartz sand with the addition of 10% SiC). As a result of the analysis carried out in-depth with the assessment of surface topography, including the use of scanning microscopy, it was possible to evaluate the influence of the parameters used on the materials studied. The obtained results, confronted with the literature data, were deepened by the hypotheses of the authors resulting from their previous studies on the phenomena of erosive wear. As a result, the following conclusions and observations have been drawn and proposed:

1. The erosive wear results confirm previous data obtained related to wear.
2. The surface topography after the conducted tests indicates clear destructive effects of the applied media.
3. Medium 1, i.e., quartz sand, is characterised by higher weight losses in the sintered samples, which indicates that it is more aggressive.
4. The addition of 10% SiC in medium 2 resulted in the appearance of areas of pronounced plastic deformation, indicating collision sites between the hard phases (SiC and WC). This state of affairs indicates that the soft matrix (Co) takes over part of the destructive energy, counteracting the erosion phenomena.
5. The soft Co matrix, as a result of contact with relatively soft particles of quartz sand, is destroyed and eroded hard WC particles are chipped off. This is due to the fact that sand particles degrade after impact, and the finest fragments penetrate the intermolecular spaces of the tested sinters.
6. The analysis of the available literature indicates a significant lack of knowledge in the area of erosive wear phenomena for carbide composites. The study of erosive wear is a preliminary study aimed at understanding the phenomena accompanying the erosive wear of sintered carbides produced by the two methods. An important aspect of these studies is the connection between wear and manufacturing conditions.
7. It is necessary to extend the study of erosive wear in the future, in order to develop a model of erosive wear of sintered materials.

## Figures and Tables

**Table 1:** Properties of the WC-Co composites consolidated by Spark Plasma Sintering, with comparisons [34].

Sample	Materials Composition	Processing Conditions	Relative Densities (100%)	Hardness HV30	Mean Fracture Toughness K_IC_ (MPam^1/2^)
Submicron	WC-6Co	SPS 1170 °C	99.26	1736 ± 38	11.3
Ultrafine	WC-4.5Co	SPS 1170 °C	99.99	1622 ± 40	12.5
Commercial	WC-4.5Co	Unknown	100.0	1705 ± 40	26.6

**Table 2:** Geometric structure parameters of the surface of samples in the initial state.

Sample	Geometrical Structure Parameters [μm]						
	S_a_	S_z_	S_v_	S_p_	S_ku_	S_sk_	S_q_
Submicron	1.02	11.61	3.62	7.99	2.29	−0.06	1.17
Ultrafine	0.81	7.03	3.03	7.99	2.29	−0.06	1.17
Commercial	0.69	7.41	4.02	3.39	3.31	−0.87	0.88

**Table 3:** Parameters of geometric structure of the surface for medium 1.

Sample	Geometrical Structure Parameters [μm]						
	S_a_	S_z_	S_v_	S_p_	S_ku_	S_sk_	S_q_
Submicron	3.36	27.93	23.91	3.02	1.18	−0.04	1.85
Ultrafine	1.62	11.75	5.62	6.13	4.86	−0.11	5.72
Commercial	2.02	14.07	7.73	6.34	1.22	−0.76	2.17

**Table 4:** Parameters of geometric structure of the surface for medium 2.

Sample	Geometrical Structure Parameters [μm]						
	S_a_	S_z_	S_v_	S_p_	S_ku_	S_sk_	S_q_
Submicron	1.81	15.02	6.13	8.89	1.97	−0.15	2.13
Ultrafine	2.05	12.03	5.68	6.35	1.99	−0.35	2.38
Commercial	1.66	11.86	7.01	4.85	2.46	−0.38	0.80

## Data Availability

Data sharing is not applicable to this article.
